# Signage-associated improvement in hand hygiene compliance: a low cost strategy

**DOI:** 10.1016/j.infpip.2022.100225

**Published:** 2022-06-03

**Authors:** Hitoshi Yamashita, Yuse Okawa, Shoko Masuyama

**Affiliations:** Graduate School of Health Sciences, Morinomiya University of Medical Sciences, 1-26-16 Nanko-Kita, Suminoe-Ku, Osaka, 559-8611, Japan

Dear Editor,

Efforts to improve healthcare workers' adherence to hand hygiene in hospitals and other healthcare facilities continue [[1], [2], [3], [4]]. Acupuncturists in Japan, as with other healthcare professionals, are expected to perform timely hand hygiene. They are also recommended to wear finger cots under certain circumstances. Although a prospective multi-centre survey conducted at educational facilities in Japan did not identify any cases of infection due to acupuncture [5], it is not possible to be certain how compliant acupuncturists are with hand hygiene procedures. One reason for this is that in most healthcare settings, acupuncture treatment is performed on a one-on-one basis in a curtained-off area. Such a situation is not ideal for promoting compliance with infection control procedures, because it is human nature to cut corners when unsupervised. Moreover, many acupuncture-related adverse events, including infection, have been reported worldwide [6]. Although not all reported events were causally related to acupuncture, strict adherence to basic infection control measures such as hand hygiene should be maintained when performing acupuncture. An optimal cost-effective strategy for improving adherence to hand hygiene recommendations would involve patients asking and monitoring the acupuncturists' behaviour [7]. However, Japanese patients generally hesitate to make comments that might make their therapists feel criticised.

Some reports suggest that there may be emotional motivators that can have a positive impact on healthcare worker behaviour and improve hand hygiene compliance [[8], [9], [10]]. Specifically, we believed that simply displaying a poster in the treatment cubicle could improve acupuncturists' hand hygiene behaviour. We tested this idea at our university's acupuncture clinic. The protocol abstract was submitted to the ‘International Forum on Quality and Safety in Healthcare 2019’ before the commencement of the study, and presented at the forum in Taipei, in September 2019.

In July 2019, we displayed a poster in each treatment cubicle at our university's acupuncture clinic, where approximately 10 acupuncturists practiced in shifts. The poster, which included photographs, informed our patients that all the acupuncturists at our university clinic use finger cots and perform the required hand hygiene procedures (Figure 1). The main outcomes measured were the changes in per-patient consumption of alcohol hand rub and finger cots, calculated based on clinic inventory records and outpatient statistics. We analysed the data in groups of 3 months (3 months = 1 term) because the monthly inventory is recorded as the number of large boxes of alcohol hand rub and finger cots, and does not change significantly from month to month.

**Figure 1:**
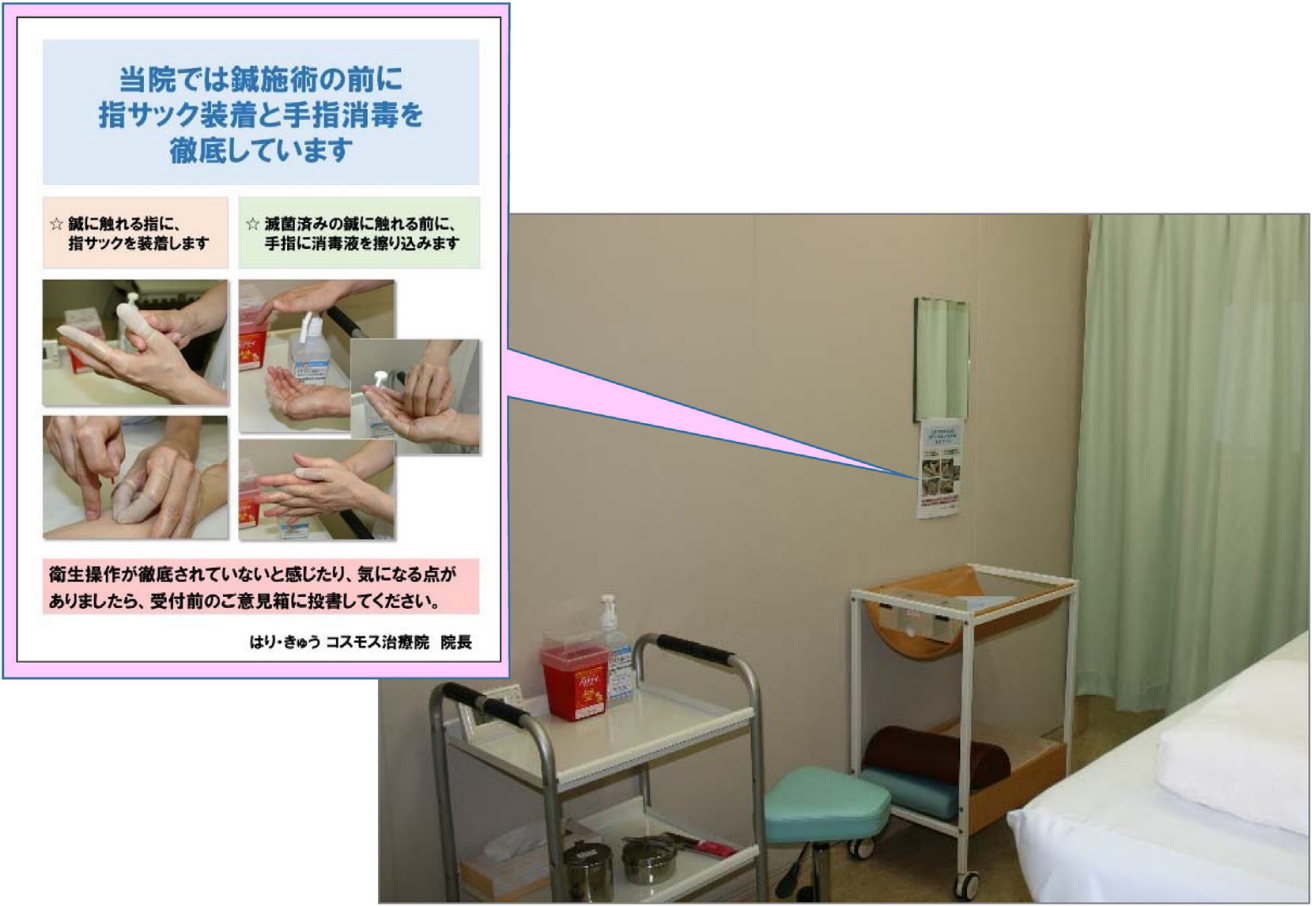
Poster displayed in a treatment cubicle.

The 1-year average consumption of alcohol hand rub and the number of finger cots used immediately prior to the poster being displayed were, 2.7 mL per patient and 2.1 per patient, respectively. After displaying the poster, the use of alcohol hand rub increased to 3.4 mL per patient in the first term and 3.1 mL per patient in the second term, while the use of finger cots decreased in both terms (Figure 2). In the third term, the use of both alcohol hand rub and finger cots returned to the baseline level. In April 2020, the clinic was temporarily closed to prevent the spread of COVID-19. Although the clinic reopened in June, we did not continue this study because the healthcare professionals' awareness of infection control procedures would have increased and may have resulted in bias.

**Figure 2:**
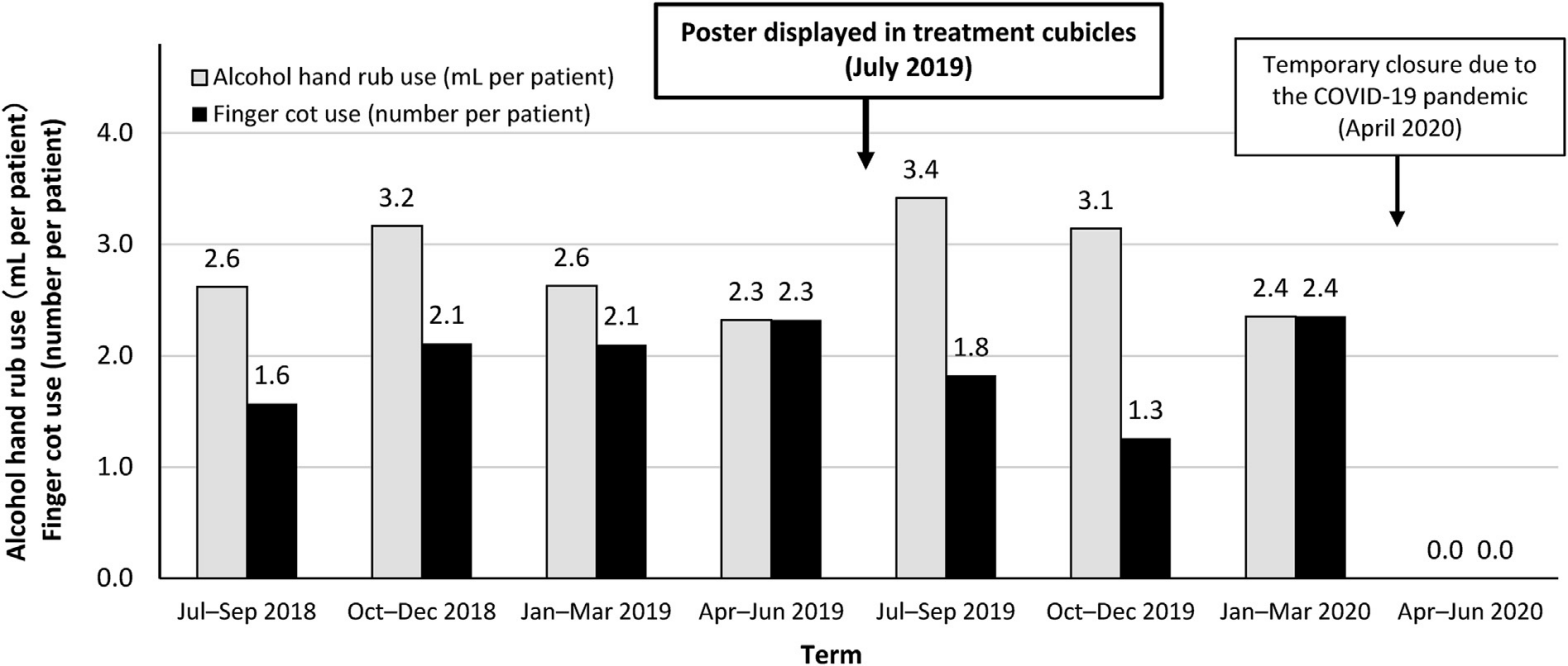
Changes in per-patient consumption of alcohol hand rub and finger cots before and after displaying the poster.

Thus, after putting up the posters, the acupuncturists' hand hygiene behaviour in terms of alcohol hand rub use improved for a maximum of 6 months. However, the use of finger cots simultaneously decreased. An explanation for this might be that the acupuncturists did not change the finger cots during a treatment session because they felt that they had disinfected their hands with alcohol hand rub more often and carefully than usual. Although there is room for improvement in compliance and the need for more rigorous studies that include a control group, displaying a poster may be an effective tool for improving healthcare professionals' adherence to hand hygiene procedures, particularly regarding the use of alcohol hand rub.

While the poster appeared to contain a message for the patients visiting the clinic, it actually targeted the acupuncturists, who inevitably saw it during every treatment session. The therapists might have been motivated by their patients' opinions of their compliance with hand hygiene. In Japan, this simple and extremely low-cost strategy would be more effective at least for a short period, rather than encouraging patients to speak up without hesitation to their therapists and caregivers.

## Conflicts of interest statement

No conflicts of interest related to this study and letter.

## Ethics approval

This study was approved by the Ethics Committee of Morinomiya University of Medical Sciences (ID number: 2019-028).

## Financial support

None.
